# A Novel Motif Identified in Dependence Receptors

**DOI:** 10.1371/journal.pone.0000463

**Published:** 2007-05-23

**Authors:** Gabriel del Rio, Darci J. Kane, Keith D. Ball, Dale E. Bredesen

**Affiliations:** 1 Buck Institute for Age Research, Novato, California, United States of America; 2 Department of Neurology, University of California San Francisco, San Francisco, California, United States of America; Baylor College of Medicine, United States of America

## Abstract

Programmed cell death signaling is a critical feature of development, cellular turnover, oncogenesis, and neurodegeneration, among other processes. Such signaling may be transduced via specific receptors, either following ligand binding—to death receptors—or following the withdrawal of trophic ligands—from dependence receptors. Although dependence receptors display functional similarities, no common structural domains have been identified. Therefore, we employed the Multiple Expectation Maximization for Motif Elicitation and the Motif Alignment and Search Tool software programs to identify a novel transmembrane motif, dubbed dependence-associated receptor transmembrane (DART) motif, that is common to all described dependence receptors. Of 3,465 human transmembrane proteins, 25 (0.7%) display the DART motif. The predicted secondary structure features an alpha helical structure, with an unusually high percentage of valine residues. At least four of the proteins undergo regulated intramembrane proteolysis. To date, we have not identified a function for this putative domain. We speculate that the DART motif may be involved in protein processing, interaction with other proteins or lipids, or homomultimerization.

## Introduction

Protein evolution is rife with examples of structural and functional domains utilized by multiple proteins: for example, over 700 human proteins display SH3 domains, and over 450 proteins display PDZ domains. The identification of such common domains may hint at similarities in substructure, interactions, and potentially in function, for the various proteins displaying these domains. About 680 different domains have been documented to date in the proteomes of humans and other organisms (SMART database, http://smart.embl-heidelberg.de/, [1]), and many of these domains appear, with some variation, in numerous proteins. Alternatively, the recognition of novel motifs may link proteins previously thought to be unrelated—e.g., suggesting a common function or interaction—and therefore may aid in the determination of both structure and function for proteins that display the novel motif.

We have previously described a set of receptors that induces apoptosis following ligand withdrawal, but inhibits apoptosis following the binding of trophic ligands [2]–[5]. These receptors have been referred to as dependence receptors. Such receptors play roles in neural development, tumorigenesis (including metastasis), neurodegeneration, and possibly in subapoptotic events such as neurite retraction and somal atrophy [2]–[5].

To date, ten such receptors have been described (Table 1 and [2]). These do not share any obvious structural similarity, nor do they display similar domains required for apoptosis induction. For example, Unc5H2 features a death domain in its intracytoplasmic region, but DCC does not; instead, apoptosis induction by DCC requires a short region in its intracytoplasmic domain (residues 1243–1264) that does not bear similarity to a death domain.

**Table 1:** Ten dependence receptors (plus orthologues) comprise the training set for the MEME query.

Entry Name	Accession #/Species	Protein Name	TM Location	DART Location	Subcellular Location	Function (per Swiss-Prot)
A4	P05067 (Human)	Amyloid beta A4 protein	700-723	705-723	Type I membrane protein	Mutated in some cases of Alzheimer's Disease
	P79307 (Pig)					
	P12023 (Mouse)					
	Q6RH29 (Canfa)					
ANDR	P10275 (Human)	Androgen receptor	690-919 (ligand-binding)	880-898	Nuclear	Regulation of eukaryotic gene expression in target tissues
	Q9GKL7 (Pig)					
	P19091 (Mouse)					
	P49699 (Rabbit)					
DCC	P43146 (Human)	Netrin receptor DCC	1098-1122	1098-1116	Type I membrane protein	Receptor for netrin required for axon guidance
	P70211 (Mouse)					
	Q63155 (Rat)					
	Q91562 (Xeno)					
PTC1	Q13635 (Human)	Patched protein homolog 1	**101-121**	101-119	Integral membrane protein	Receptor for sonic hedgehog (SHH), indian hedgehog (IHH) and desert hedgehog (DHH). May have a tumor suppressor function
	Q61115 (Mouse)		437-457	574-592		
	Q90693 (Chick)		473-493	1029-1047		
	Q09614 (Caeel)		502-522	1085-1103		
			548-568			
			**578-598**			
			749-769			
			**1028-1048**			
			1056-1076			
			**1084-1104**			
			1122-1141			
			1155-1175			
RET	P07949 (Human)	Proto-oncogene tyrosine-protein kinase receptor ret	636-657	637-655	Type I membrane protein	Part of GDNF receptor complex with tyrosine-protein kinase activity
	P35546 (Mouse)					
	Q9EPA1 (Rat)					
	O42362 (Brare)					
TNR5	P25942 (Human)	Tumor necrosis factor receptor superfamily member 5	194-215	195-213	Type I membrane protein	Receptor for TNFSF5/CD40L
	P27512 (Mouse)					
	Q28203 (Bovine)					
	Q7YRL5 (Canfa)					
TNR16	P08138 (Human)	Tumor necrosis factor receptor superfamily member 16	251-272	257-275	Type I membrane protein	Common neurotrophin receptor
	P07174 (Rat)					
	Q9Z0W1 (Mouse)					
	P18519 (Chick)					
UNC5A	Q6ZN44 (Human)	Netrin receptor UNC5A	307-327	309-327	Type I membrane protein	Receptor for netrin required for axon guidance
UNC5B	Q8IZJ1 (Human)	Netrin receptor UNC5B	378-398	381-399	Type I membrane protein (by similarity)	Receptor for netrin required for axon guidance (axon repulsion)
UNC5C	O95185 (Human)	Netrin receptor UNC5C	381-401	382-400	Type I membrane protein (by similarity)	Receptor for netrin required for axon guidance (repulsion)
	O08747 (Mouse)					

Ten dependence receptors plus their orthologues (32 sequences total) were used as a training set by the MEME program to search for high-scoring motifs common to all proteins. Table 1 shows the transmembrane location (for each protein with one) and the location of the DART motif. Data taken from the Swiss-Prot database. All accession numbers are from Swiss-Prot.

In the present study, we attempted to determine whether dependence receptors as a group may indeed display a common motif(s) that had gone undetected by the initial comparisons of alignment and predicted (known) domains. We utilized the Multiple EM (Expectation Maximization) for Motif Elicitation program (MEME) (http://meme.sdsc.edu/meme/meme.html and [6]) and identified a novel motif that is featured by receptors that have been described as dependence receptors. We then searched the Swiss-Prot protein database (http://www.expasy.uniprot.org/ and [7]) using the Motif Alignment and Search Tool (MAST) program (http://meme.sdsc.edu/meme/mast.html and [8]), to determine whether other receptors or other proteins also feature this motif, and identified an additional 16 human proteins that display this motif (see Results and Discussion, below).

The novel putative motif is in a transmembrane region, and therefore was dubbed dependence-associated receptor transmembrane motif (DART motif). Here we describe the consensus sequence and discuss the possible functions of this novel motif.

## Materials and Methods

### Databases and software

The UniProt Knowledgebase (UniProtKB; http://www.expasy.uniprot.org/) database Release 3.3 (consisting of Swiss-Prot Release 45.3 and TrEMBL Release 28.3) from the Swiss Institute of Bioinformatics was used for this study. This database was chosen because it is well documented and allowed us to analyze the predictions on receptors.

The MEME (http://meme.sdsc.edu/meme/meme.html) software program (version 3.0; non-commercial version) was used for the identification of motifs in non-aligned sequences, where a motif is a sequence pattern that occurs repeatedly in a group of protein or DNA sequences. MEME saves these motifs as a set of profiles. MEME uses the method of Bailey and Elkan to identify likely motifs within the input set of sequences [6]. A range of motif widths (>15 amino acids in length) and various numbers of unique motifs to search for (zero or one motifs per sequence) were specified in our queries.

The software program MAST (http://meme.sdsc.edu/meme/mast.html, version 3.0; non-commercial version) was used to search the Swiss-Prot database for other proteins displaying the motifs identified by MEME to be present in more than one dependence receptor. The algorithm in MAST calculates position scores for each profile at each possible position within a sequence. These scores are translated into p-values, which represent the likelihood of the given profile scoring that well against a randomly generated sequence. The best (i.e., lowest) position p-values for each profile are then adjusted to take into account the length of the sequence. MAST avoids allowing gaps in the profiles or in the search sequence.

### Training Set

In order to search for motifs in previously described dependence receptors, we used a set of ten human receptors and their corresponding orthologues (three orthologues were used for each, in order to avoid bias generated by using more orthologues for one dependence receptor than another) found in the UniProtKB database. Thus, our training set included a total of 32 protein sequences (32 rather than 40, since the netrin receptors Unc5A, Unc5B, Unc5C were represented by a total of 4 sequences rather than 12, to prevent overweighting). This list is shown in Table 1.

The option of having the training set sequences “shuffled” provided one of the controls used, ensuring that the motif(s) we detected were significant.

### Web Site

To maintain updated information on the dependence receptor field and to allow researchers to identify the DART motif in their protein of interest, we have developed a website (http://bis.ifc.unam.mx/DependenceReceptors/). The program at our website runs four independent predictions: three for identifying a transmembrane region and one for identifying the DART motif. The transmembrane region predictions are run through three different programs located at:

1. HMMTOP (http://www.enzim.hu/hmmtop/),
2. SOSUI (http://sosui.proteome.bio.tuat.ac.jp/sosui_submit.html) and
3. TMPRED (http://www.ch.embnet.org/software/TMPRED_form.html).

For identifying the DART motif, the website uses the MAST program.

## Results

### Searching for motifs among the known dependence receptors

The dependence receptors listed in Table 1 have all been shown to induce programmed cell death when expressed in the absence of their respective trophic ligands, but not when bound by these same trophic ligands. The receptors' non-orthologous sequences in the training set did not show any significant sequence similarity by simple alignment searches. Hence, in order to search for novel motifs in this set, we used the MEME program. MEME allows the identification of motifs in non-aligned sequences, where a motif is a sequence pattern that occurs repeatedly in a group of protein or DNA sequences (see Materials and Methods section). Although MEME has been most commonly used to identify motifs in homologous sequences, a pattern identified by MEME in non-homologous sequences may be biologically relevant if: a) the proteins in the training set share a common function and b) the proteins identified to contain the motif could reasonably be suspected to share functional features with the training set. Note that these are the same conditions that are considered when evaluating motifs identified in homologous proteins.

Using a training set consisting of 32 sequences from the 10 experimentally-proven dependence receptors (Table 1), we identified a novel motif that occurs in all of the training set proteins. This motif, designated “DART” (dependence-associated receptor transmembrane) motif, appeared in the transmembrane region of all proteins in the training set that include a transmembrane region, whereas it appeared in the ligand-binding region of the one protein that lacks a transmembrane region (the androgen receptor).

**Figure 1:**
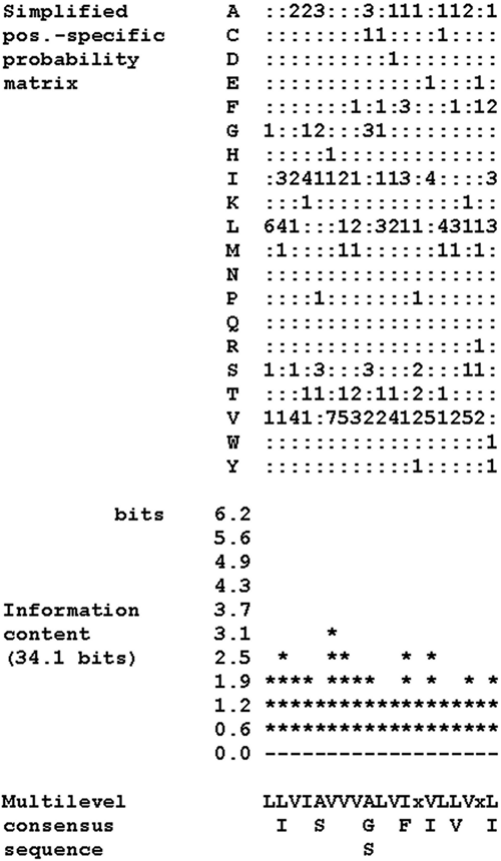
Multilevel consensus sequence and amino acid frequency of the DART motif. MEME motifs are represented by position-specific probability matrices that specify the probability of each possible letter appearing at each possible position in an occurrence of the motif. In order to make it easier to see which letters are most likely in each of the columns of the motif, the simplified motif shows the letter probabilities multiplied by 10 rounded to the nearest integer. Zeros are replaced by “:” (a colon) for readability. The information content diagram provides an idea of which positions in the motif are most highly conserved. Each column (position) in a motif can be characterized by the amount of information it contains (measured in bits). Highly conserved positions in the motif have high information; positions where all letters are equally likely have low information. The diagram is printed so that each column lines up with the same column in the simplified position-specific probability matrix above it. This multilevel consensus sequence says several things about the motif. First, the most likely form of the motif can be read from the top line as LLVIAVVVALVIxVLLVxL. Second, that only letter L has probability more than 0.2 in position 1 of the motif, both L and I have probability greater than 0.2 in position 2, etc. Third, a rough approximation of the motif can be made by converting the multilevel consensus sequence into the Prosite signature: L-[LI]-V-I-[AS]-V-V-V-[AGS]-L-V-[IF]-x-[VI]-L-[LV]-V-x-[LI].

**Figure 2:**
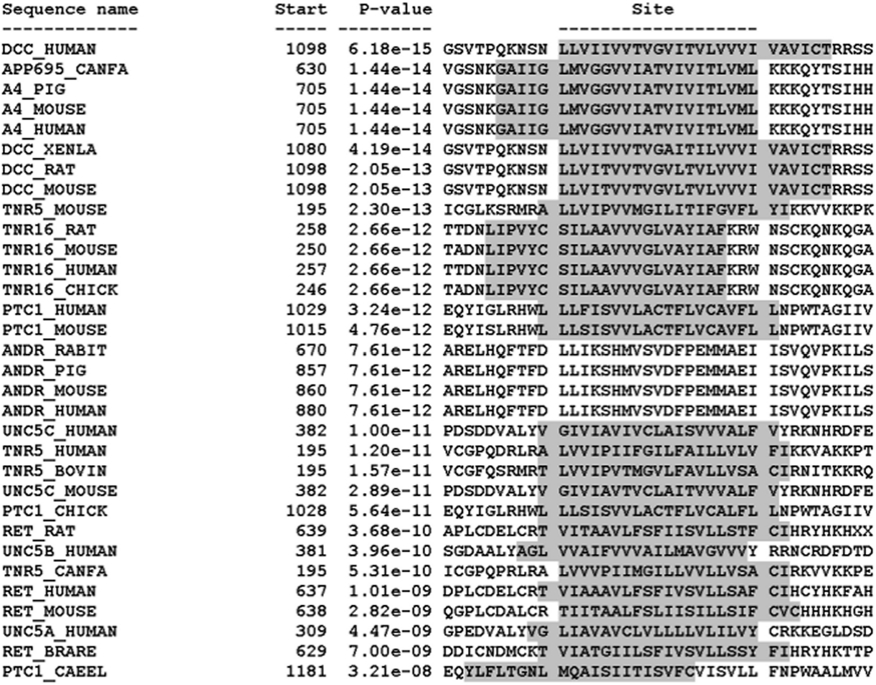
Aligned DART motif within all dependence receptor training set members. Occurrences (sites) of the DART motif within the sequences of the 32 dependence receptors that were used as the training set. The sites are shown aligned with each other, and the ten sequence positions preceding and following each site are also shown. Each site is identified by the name of the sequence where it occurs and the position in the sequence where the site begins. The sites are listed in order of increasing p-value (decreasing statistical significance). The p-value of a site is computed from the match score of the site with the position specific scoring matrix for the motif. The p-value gives the probability of a random string (generated from the background letter frequencies) having the same match score or higher. Amino acid residues constituting the transmembrane region of the protein are indicated by shading.

The consensus sequence of the proposed DART domain is shown in Figure 1, and the DART motifs from the training set proteins are aligned in Figure 2.

**Figure 3:**
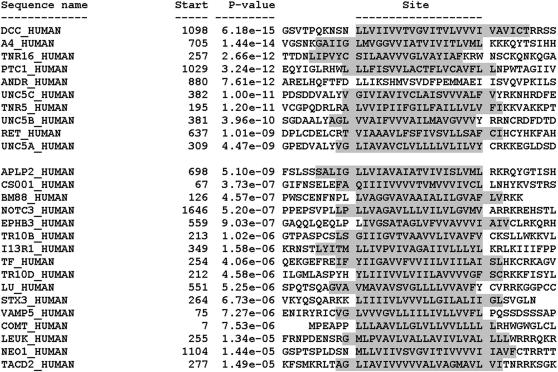
Aligned DART motif within 26 human proteins. Occurrences (sites) of the DART motif within the sequences of the 10 human dependence receptors from the training set (top 10 of list) and the 16 human proteins designated by MAST as containing the motif. The sites are shown aligned with each other, and the ten sequence positions preceding and following each site are also shown. Each site is identified by the name of the sequence where it occurs and the position in the sequence where the site begins. The sites are listed in order of increasing p-value. The p-value of a site is computed from the match score of the site with the position specific scoring matrix for the motif. The p-value gives the probability of a random string (generated from the background letter frequencies) having the same match score or higher. Amino acid residues constituting the transmembrane region of the protein are indicated by shading.

**Figure 4:**
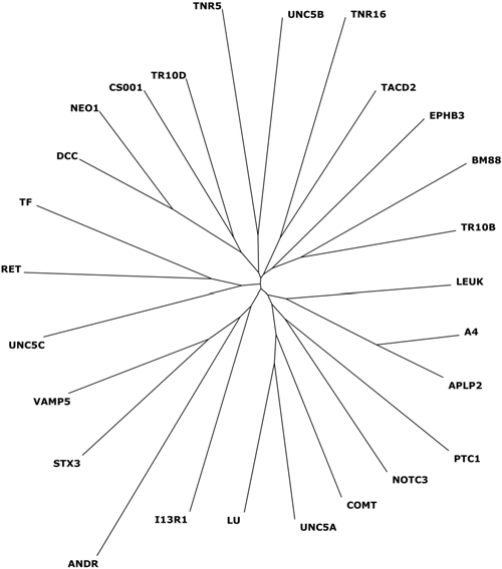
Dendrogram of the 26 human DART-containing proteins. Dendrogram demonstrating the relationships of the DART motif sequences within each of the 26 human proteins found to contain DART (10 from the training set plus 16 discovered through the use of MAST).

Using the software program MAST to search the Swiss-Prot database for other proteins that displayed the DART motif, we found an additional 54 sequences, 16 of which are human proteins (using a cut-off at E-value of 2.7, the value below which all training set members scored) (Table 2). Of 13,991 human proteins in the database, 3,465 are annotated as transmembrane proteins, and 25 display the DART motif—nine of the 10 training set members (the exception being, as noted above, the androgen receptor) and 16 additional human proteins (Table 3). Of these 16 additional proteins, all were transmembrane proteins, and all contained the DART motif within their transmembrane region. Thus the DART motif is relatively uncommon (at least as defined here), occurring in approximately 0.7% of human transmembrane proteins (25/3465). If we include slightly less similar motifs, extending the acceptable E-value from 2.7 to 10, then an additional 4 human proteins are included (data not shown). The alignment of the putative DART domains of these 16 human non-training set proteins is shown in Figure 3. A dendrogram of the human DART motifs is shown in Figure 4.

**Table 2:** MAST result list of 54 non-training set proteins found to display the DART motif.

Swiss-Prot Accession # / Sequence Name	Protein Name (truncated)
sp|Q05910|ADAM8_MOUSE	ADAM 8 precursor (A disin…
sp|Q06335|APLP2_MOUSE	Amyloid-like protein 2 pr…
sp|Q06481|APLP2_HUMAN	Amyloid-like protein 2 pr…
sp|P15943|APLP2_RAT	Amyloid-like protein 2 pr…
sp|Q9JKC6|BM88_MOUSE	BM88 antigen
sp|Q8N111|BM88_HUMAN	BM88 antigen
sp|Q29026|BM88_PIG	BM88 antigen
sp|P21964|COMT_HUMAN	Catechol O-methyltransfer…
sp|Q12215|WSC3_YEAST	Cell wall integrity and s…
sp|P22521|IMMV_ECOLI	Colicin V immunity protei…
sp|P18466|HA19_CANFA	DLA CLASS I HISTOCOMPATIB…
sp|P54753|EPHB3_HUMAN	Ephrin type-B receptor 3 …
sp|Q07498|EPHB3_CHICK	Ephrin type-B receptor 3 …
sp|Q91735|EPHB3_XENLA	Ephrin type-B receptor 3 …
sp|P54761|EPHB4_MOUSE	Ephrin type-B receptor 4 …
sp|P04855|FUS_SENDZ	Fusion glycoprotein F0 pr…
sp|P18345|VGLE_EHV4	Glycoprotein E
sp|P32343|YKM4_YEAST	Hypothetical 65.1 kDa pro…
sp|O13785|YEO7_SCHPO	Hypothetical protein C17G…
sp|Q21874|YF1M_CAEEL	Hypothetical protein R09E…
sp|Q83S36|YBHG_SHIFL	Hypothetical UPF0194 memb…
sp|Q8FJN6|YBHG_ECOL6	Hypothetical UPF0194 memb…
sp|P75777|YBHG_ECOLI	Hypothetical UPF0194 memb…
sp|Q8X7Y9|YBHG_ECO57	Hypothetical UPF0194 memb…
sp|P78552|I13R1_HUMAN	Interleukin-13 receptor a…
sp|P16150|LEUK_HUMAN	Leukosialin precursor (Le…
sp|P50895|LU_HUMAN	Lutheran blood group glyc…
sp|P32334|MSB2_YEAST	MSB2 protein (Multicopy s…
sp|Q9HZL1|NQRF_PSEAE	Na(+)-translocating NADH-…
sp|P97798|NEO1_MOUSE	Neogenin precursor
sp|Q92859|NEO1_HUMAN	Neogenin precursor
sp|Q9UM47|NOTC3_HUMAN	Neurogenic locus notch ho…
sp|P46697|PPIB_MYCLE	Probable peptidyl-prolyl …
sp|P50605|SDC_CAEEL	Probable syndecan precurs…
sp|P20990|VA13_VACCC	Protein A13
sp|P33838|VA13_VARV	Protein A13
sp|O15165|C181_HUMAN	Protein C18orf1
sp|Q53902|MMLA_STRCO	Putative membrane protein…
sp|P06494|ERBB2_RAT	Receptor tyrosine-protein…
sp|Q8E6G4|EZRA_STRA3	Septation ring formation …
sp|P49415|SDC_DROME	Syndecan precursor
sp|P26260|SDC1_RAT	Syndecan-1 precursor (SYN…
sp|P18828|SDC1_MOUSE	Syndecan-1 precursor (SYN…
sp|Q64704|STX3_MOUSE	Syntaxin-3
sp|Q08849|STX3_RAT	Syntaxin-3
sp|Q13277|STX3_HUMAN	Syntaxin-3
sp|P13726|TF_HUMAN	Tissue factor precursor (…
sp|Q9D7R2|TMEPA_MOUSE	Transmembrane prostate an…
sp|O14763|TR10B_HUMAN	Tumor necrosis factor rec…
sp|Q9UBN6|TR10D_HUMAN	Tumor necrosis factor rec…
sp|P09758|TACD2_HUMAN	Tumor-associated calcium …
sp|O70404|VAM8_MOUSE	Vesicle-associated membra…
sp|Q9WUF4|VAM8_RAT	Vesicle-associated membra…
sp|O95183|VAM5_HUMAN	Vesicle-associated membra…

The top-scoring non-training-set proteins displaying the DART motif, representing 38 proteins (plus 16 orthologues). Sixteen of the 54 are the human proteins listed in Table 3.

**Table 3:** 16 human proteins found to display the DART motif.

Entry Name	Accession #	Protein Name	TM Location	DART Location	Subcellular Location	Function (per Swiss-Prot)
APLP2	Q06481	Amyloid-like protein 2 [Precursor]	693-716	698-716	Type I membrane protein (MP)	May play a role in the regulation of hemostasis
CS001	O15165	Protein C18orf1	65-85	67-85	Type Ib MP	May confer susceptibility to schizophrenia
BM88	Q8N111	BM88 antigen	126-146	126-144	Type IV MP	Involved in neuroblastoma cell differentiation (by similarity)
NOTC3	Q9UM47	Neurogenic locus notch homolog protein 3	1644-1664	1646-1664	Type I MP	Receptor for membrane-bound ligands Jagged1, Jagged2 and Delta1 to regulate cell-fate determination
EPHB3	P54753	Ephrin type-B receptor 3	560-580	559-577	Type I MP	Receptor for members of the ephrin-B family
TR10B	O14763	Tumor necrosis factor receptor superfamily member 10B	211-231	213-231	Type I MP	Receptor for TNFSF10/TRAIL
I13R1	P78552	Interleukin-13 receptor alpha-1 chain	344-367	349-367	Type I MP	Binds IL13 with a low affinity
TF	P13726	Tissue factor	252-274	254-272	Type I MP	Initiates blood coagulation by forming a complex with circulating factor VII or VIIa
TR10D	Q9UBN6	Tumor necrosis factor receptor superfamily member 10D	212-232	212-230	Type I MP	Receptor for TRAIL
LU	P50895	Lutheran blood group glycoprotein	548-568	551-569	Type I MP	Probable receptor. May mediate intracellular signaling. Member of the immunoglobulin superfamily IG
STX3	Q13277	Syntaxin-3	264-284	264-282	Type IV MP	Potentially involved in docking of synaptic vesicles at presynaptic active zones
VAMP5	O95183	Vesicle-associated membrane protein 5	73-93	75-93	Type IV MP	May participate in trafficking events that are associated with myogenesis
COMT	P21964	Catechol O-methyltransferase	7-26	7-25	Type II MP	Catalyzes the O-methylation of catecholamine neurotransmitters and catechol hormones
LEUK	P16150	Leukosialin	254-276	255-273	Type I MP.	Physicochemical properties of the T-cell surface and lectin binding
NEO1	Q92859	Neogenin	1106-1126	1104-1122	Type I MP	Receptor for repulsive guidance molecule
TACD2	P09758	Tumor-associated calcium signal transducer 2	275-297	277-295	Type I MP	May function as growth factor receptor

Sixteen human proteins were discovered that display high-scoring matches for the DART motif when the Swiss-Prot database was searched using the MAST software program. Table 3 shows the transmembrane location and the location of the DART motif. Data taken from the Swiss-Prot database. All accession numbers are from Swiss-Prot.

### Structure of the putative DART domain

**Figure 5:**
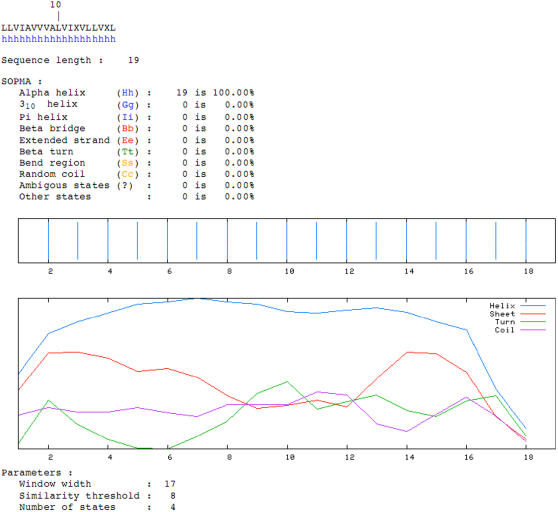
Predicted secondary structure of the consensus sequence of DART. SOPMA analysis demonstrates the alpha-helical nature of the putative DART domain.

The predicted secondary structure of the consensus DART domain, as predicted by the SOPMA (Self Optimized Prediction Method from Alignments) method [9] is for a helical structure (Figure 5). This is not surprising, given that the motif lies within the transmembrane region of the proteins that display it. However, in comparison to other transmembrane regions (in randomly selected transmembrane proteins) it is valine rich (29±4% vs. 15±3%; p<0.001).

## Discussion

The function of this novel motif is currently unknown. The finding that it exists in all dependence receptors described to date suggests that it may play a role in some biochemical process related to their function, such as the induction of apoptosis or the inhibition of apoptosis following ligand binding, or possibly an interaction with another membrane protein or membrane-associated non-proteinaceous molecule such as a lipid. For at least three of the proteins in the training set—APP, p75NTR, and DCC—this region undergoes regulated intramembrane proteolysis (RIP) [10], releasing an intracytoplasmic fragment that may migrate to the nucleus. Thus it is possible that the other proteins that display the DART motif may be substrates that also undergo regulated intramembrane proteolysis; however, a number of proteins that have been shown to undergo such cleavage do not display a DART motif, so it is clearly not required for such processing.

It is noteworthy that the transmembrane regions of DART-containing proteins are valine rich, with nearly twice the percentage of valine residues present in randomly-selected human Type I transmembrane protein domains (29±4% vs. 15±3%; p<0.001). It has been shown that Leu heptads within transmembrane domains may serve as homomultimerization domains and that the substitution of Ala (or other residues typical of transmembrane regions, including Val) for Leu may prevent homomultimerization [11]. Thus one possibility for the Val-rich nature of the DART domain may relate to the inhibition of receptor homomultimerization.

### Proteins identified by MAST as displaying the DART motif

As noted above, MAST identified 16 proteins, all transmembrane proteins displaying the DART motifs and were as similar to the consensus as those of the training set (Table 3; Figure 3). Most of these have been implicated in cell death, either directly or indirectly; furthermore, several bind trophic ligands, as well, making them reasonable candidates to evaluate experimentally as possible dependence receptors.

Neogenin has recently been shown to bind RGM (repulsive guidance molecule), and to serve as a possible dependence receptor for RGM, inducing programmed cell death that is inhibited by RGM [12]. Therefore, the identification of a DART motif within neogenin provides further support for RGM as a candidate dependence receptor.

APLP2 (APP-like protein 2) has been shown previously to be similar to APP in displaying a potential caspase-cleavage site in its intracytoplasmic domain [13]. Cleavage at this site liberates a pro-apoptotic peptide, C31, similar to what has been demonstrated for APP. Thus, although it is not yet clear whether APLP2 functions as a dependence receptor, and in particular whether APLP2 binds a trophic ligand, by analogy to APP it may bind laminin, collagen IV, glypican, or another ligand [14]–[16], and thus serve as a dependence receptor for one or more of those ligands.

Notch is an extensively-studied transmembrane receptor involved in cell fate determination. It binds to ligands Delta1, Jagged1, and Jagged2, regulating differentiation, proliferation, and apoptosis. Notch, like APP, DCC, and p75NTR, undergoes regulated intramembrane proteolysis, liberating an intracytoplasmic domain, the NICD, that forms a transcriptional activator complex with RBP-J kappa, activating genes of the enhancer of split locus.

Ephrin type B receptor 3 binds both ephrin-B1 and ephrin-B2. It is not yet known whether this receptor induces programmed cell death in the absence of ephrin-B1 or –B2 binding.

Tumor-associated calcium signal transducer 2 (TACD2) may function as a trophic factor receptor, but its ligand is currently unknown.

Three of the proteins identified by MAST as displaying a DART motif are involved in neurotransmitter synthesis or release. Catechol O-methyltransferase exists in both cytosolic and membrane-spanning (type II membrane protein) forms, and this latter displays a DART motif. Syntaxin-3 is a Type IV membrane protein potentially involved in docking of synaptic vesicles at presynaptic active zones. Vesicle-associated membrane protein 5 (VAMP5) is also a Type IV membrane protein that may participate in vesicle trafficking events associated with myogenesis.

### Conclusion

Ten of ten previously described dependence receptors display a region of similarity dubbed the DART (dependence-associated receptor transmembrane) motif. MAST identified this motif in an additional 16 human proteins in the SwissProt database, in all cases in the transmembrane regions. The function of this novel putative domain is unknown, but the motif is noted to be valine rich, and in at least four cases, the DART motif is a site of regulated intramembrane proteolysis (RIP). Whether or not this motif plays a functional role in cell death induction or ligand-induced inhibition mediated by dependence receptors remains to be determined, but the identification of this motif in 16 non-training-set proteins such as Notch and APLP2 raises the question of whether these proteins may also function as dependence receptors. Since the field of dependence receptors is an emerging field, we have developed a website for predicting dependence receptors at http://bis.ifc.unam.mx/DependenceReceptors/.
